# Man-marking, pressure on the ball, and offside and their effect on physiological, physical, technical and tactical parameters during small-sided games

**DOI:** 10.3389/fspor.2026.1733632

**Published:** 2026-01-29

**Authors:** Michael C. Rumpf, Johannes Jäger, Aaron Uthoff, Matthias Lochmann

**Affiliations:** 1 Department of Sport Science and Sport, Friedrich-Alexander University Erlangen-Nürnberg, Erlangen, Germany; 2 Footballscience.net, Dreieich, Germany; 3 Sport Performance Research Institute New Zealand, AUT-University, Auckland, New Zealand

**Keywords:** football, man-marking, offside, pressure on the ball, soccer, training

## Abstract

Man-marking (MM), and pressure on the ball are task constraints frequently manipulated by coaches during small-sided games (SSG). Man marking seemed to increase the physiological load of SSG measured through heart rate measures [average and percentage heart rate (HR), time spent >80% maximum HR] and rating of perceived exertion (RPE). Additionally, MM increased the physical parameters in soccer players. Total distance covered, as well as the relative distance and other related variables with higher intensity such as distance in velocity bands at high-intensity >13 km/h as well as sprinting increased in comparison to non-MM. Players also experienced greater work rate, as reported through the work-to-rest ratio. Pressure on the ball increased players’ physiological intensity (e.g., percentage of time spent in 90%–100% HR max) and physical load (e.g., number and distance of high-speed runs). Tactical changes through pressure on the ball was expressed as higher number of ball recovery, lower decision making and execution with a lower spatial exploration index. Utilizing offside during SSG did not change the physiological load of players whilst decreasing total distance and low(er) speed running such 7–16 km/h). Offside affected players depth positioning, consequently the length-per-width ratio and players’ spatial exploration. As a result, coaches can utilize MM and pressure on the ball to increase intensity of SSG. Offside, however, diminishes physicality of games whilst restricting tactical behaviour of players.

## Introduction

The effectiveness of small-sided games (SSG) depends on a range of configuration variables, such as the composition of teams, the number of players per team, the size of the pitch, and the manipulation of the game rules (1). Examples of SSG rule modifications include altering the field dimensions (2) and relative pitch size (3), restricting the number of touches (4), playing with or without the goalkeeper (5), implementing man-marking (6, 7), and applying pressure on the ball (8). Numerous reviews have examined the influence of these task constraints on physical, technical, and tactical characteristics during SSG (6, 9). However, despite the extensive focus on physical outcomes, there remains a limited understanding of how manipulations such as man-marking (MM) and pressure on the ball affect technical and tactical aspects of play. This gap in the literature is noteworthy, as MM is a specific type of defense strategy (10) utilized to slow down the attack (11) and is frequently performed across different player positions (12). Similarly, pressure on the ball is exerted by defending players (13) and was defined as a vital component in transitional phases (14).

Recent developments in soccer have shown an increasing emphasis on applying higher defensive pressure and pressing further up the field at a faster tempo (15). This strategy enables teams to regain possession quickly and initiate coordinated counterattacks, with the primary objective of creating scoring opportunities from the zone where possession was recovered (16). Therefore, pressing seems to be a key factor in modern soccer performance, and training approaches that replicate these game demands are essential for developing tactical awareness, physical readiness, and decision-making under pressure.

The offside rule remains one of the most controversial aspects of football (17) and requires officials to make frequent, high-consequence decisions (18), especially given evidence of the existence of home team bias in officiating (19). Across professional competitions, the average number of offside calls per match appears to have declined over time, from approximately 4.3–6.3 in 2009/2010 to 3.1 to 4.5 in 2018/2019 (20). Over this period, referees also work to improve the accuracy of offside decisions (21). Because offside situations can affect match outcomes and discriminated winning from losing teams (22), coaches apply the rule during SSG in the training process.

Understanding how the aforementioned task constraints influence physiological, physical, technical and tactical performance outcomes is therefore critical for optimising SSG design and ensuring that training transfers effectively to match play. Accordingly, this mini review aims to discuss the influence of man-marking, pressure on the ball, and the offside rule on physiological, physical, technical, and tactical parameters during SSG in soccer. By synthesising existing evidence, the mini-review seeks to clarify the extent to which these task constraints contribute to game realism and player development, while identifying gaps for future research and practical application.

## Methods

Major databases (PubMed, Web of Science, SportDiscus, and Scopus, Google scholar) were searched from earliest available until September 2025 with the following key words “coaching”, “training”, “offside”, “soccer”, “football”, “small-sided games”, “pressure on the ball”, “tactical instructions” and “man-marking”. This search was supplemented by utilizing grey literature (https://opengrey.eu) and reference list screening of all included studies, along with searches within previous reviews on similar topics. Identification of suitable articles involved the initial screening of the titles and abstracts while the second stage comprised a full-text review of the remaining articles. To be included in this review, studies needed to focus on man-marking, pressure on the ball and offside during small-sided games and its effect on physiological, physical, technical and tactical parameter. Nevertheless, Table 1 displays all references and the effect of the condition on outcome variables.

**Table 1:** All references, divided by condition type and the effect of on outcome variables.

Reference	Population	Playing format	Set-up protocol	Outcome in variable as per condition
Man-marking				
Cihan et al. (16)	*N* = 18, male youth 19.6 years of age, national elite academy	3 vs. 3 2 vs. 2 + 2	Relative pitch size: 116 m^2^ Protocol: 3 × 4 min with 5 min passive recovery	Physiological variables: HRMean ↑, % HRMax ↑, RPE ↑, blood lactate ↑ Physical variables: Total distance ↑, distance covered >18 km/h ↑, distance covered 13.0–17.9 km/h ↑
Aroso et al. (27)	*N* = 14, male youth 14-15 years of age, national standard	a) 2 vs. 2 b) 3 vs. 3 c) 4 vs. 4	Relative pitch size: a) 150 m^2^, b) 100 m^2^, c) 75 m^2^ Protocol: a) 3 × 90 sec with 90 sec passive recovery b) 3 × 4 min with 90 sec passive recovery c) 3 × 6 min with 90 sec passive recovery	Physiological variables: blood lactate ↑ Physical variables: Time walking ↑, lateral and backwards running ↑
Ngo et al. (28)	*N* = 12, male youth 16.2 years of age, 4 hours soccer training per week	3 vs. 3	Relative pitch size: 75 m^2^ Protocol: 3 × 4 min with 4 min passive recovery	Physiological variables: % HRReserve ↑, RPE ↑ (only when goals were included)
Sampaio et al. (29)	*N* = 8, male youth 15 years of age, national standard	a) 2 vs. 2 b) 3 vs. 3	Relative pitch size: a) 150 m^2^, b) 100 m^2^ Protocol: a) 2 × 3 min with 90 sec passive recovery b) 2 × 3 min with 90 sec passive recovery	Physiological variables: RPE ↑
Aasgaard et al. (30)	*N* = 8, male adult 23.6 years of age, national standard	a) 2 vs. 2 b) 3 vs. 3 c) 4 vs. 4	Relative pitch size: a) 100 m^2^, b) 105 m^2^, c) 108 m^2^ Protocol: 4 × 4 min with 2 min passive recovery	Physiological variables: % HR ↑, RPE ↑ Physical variables: Total distance ↑, relative total distance ↑, average speed ↑, sprint distance ↑, distance covered > 18 km/h ↑
Casamichana et al. (31)	*N* = 18, male adult 23.4 years of age, amateur	a) 3 vs. 3 b) 6 vs. 6 c) 9 vs. 9	Relative pitch size: ∼92 m^2^ Protocol: 4 × 4 min with 2 min passive recovery	Physical variables: Total distance ↑, player load ↑, work-to-rest ratio ↑
Pressure on the ball				
Chen et al. (8)	*N* = 48, male youth 15.4 years of age, from four professional clubs	2 vs. 2	Relative pitch size: 113 m^2^ Protocol: 5 × 2 min with 4 min passive recovery	Physiological variables: HRMean ↑, % time spent in 90%–100% HRMax ↑, blood lactate ↑, % time spent in 80%–90% HRMax ↓ Physical variables: Number of high-speed runs ↑, Number of direction changes ↑, relative high-speed running (20–25 km/h) distance (m/min) ↑, relative moderate-speed running (15–20 km/h) distance (m/min) ↑ Tactical variables: Number of ball recoveries ↑
Cihan et al. (16)	*N* = 18, male youth 19.6 years of age, national elite academy	3 vs. 3 2 vs. 2 + 2	Relative pitch size: 116 m^2^ Protocol: 3 × 4 min with 5 min passive recovery	Physiological variables: HRMean ↑, % HRMax ↑, session-RPE ↑, blood lactate ↑ Physical variables: Total distance ↑, distance covered >18 km/h ↑, distance covered 13.0–17.9 km/h ↑
Offside				
Custodio et al. (42)	*N* = 24, male youth 16.7 years of age, national league players with 7 training sessions per week	3 vs. 3	Relative pitch size: 162 m^2^ Protocol: 4 × 4 min with 5 min passive recovery	Physiological variables: HRMean NC, % HRMax NC
Castillo et al. (40)	*N* = 24, male youth 11.8 years of age, three training sessions/week	6 vs. 6	Relative pitch size: 25 m^2^, 50 m^2^, 75 m^2^ Protocol: 6 min	Physical variables: Total distance ↓, distance covered 13.0–16.0 km/h ↓
Custodio et al. (43)	*N* = 24, male youth 16.7 years of age, national league players with 7 training sessions per week	3 vs. 3	Relative pitch size: 162 m^2^ Protocol: 4 × 4 min with 5 min passive recovery	Physical variables: Total distance ↓, average speed ↓, distance covered 7.0-12.9 km/h ↓, distance covered 13.0-18.0 km/h ↓, distance covered 0.0–6.9 km/h ↑
Praca et al. (42)	*N* = 24, male youth 16.7 years of age, national-level competition, 7 training sessions per week	3 vs. 3	Relative pitch size: 162 m^2^ Protocol: 4 × 4 min with 5 min passive recovery	Tactical variables: Playing length ↓, length-per-width ratio ↓, spatial exploration ↓

RPE, rating of perceived exertion; %, percentage; max, maximum; ↑, significant increase; ↓, significant decrease.

### Man marking

Soccer players are coached to structure their behavior based on strategic plans that include specific defensive tactics designed to limit the opponent's attacking opportunities (23). For example, MM is frequently used during set-pieces (24) and was one of specific actions besides high pressing and low-block formations designed to either disrupt the opponent's build up play early or maintain compactness within the defensive third (23). This defensive strategy has been associated with higher player density, resulting in elevated physiological strain, such as higher blood lactate concentrations and increased fatigue compared with zonal defensive strategies (25). Consequently, coaches have implemented MM in SSG formats across varying age groups and formats, including youth (26–29) and adult players (30) in 2 vs. 2 (27, 29, 30), 3 vs. 3 (26–30) and 4 vs. 4 (27, 30) configurations.

### Man marking on physiological parameters

Man marking appears to increase the physiological intensity of SSG, particularly in formats involving two to four players per team. Studies (*n* = 6; 78 players) have reported higher average heart rate (HR) (26), percentage (%) of HR (26, 28, 30), time spent >80% HR (31), rating of perceived exertion (RPE) (26, 28–30), and blood lactate concentration (26, 27) when MM is employed. Interestingly, MM increased HR measures irrespective of other task constraints, such as game objective (possession play vs. goal scoring) (28). However, significant increases in session RPE were only evident when games included goals (28). Player number also appears to influence the physiological response, with average HR% (30) and time spent > 80% max HR being higher in the small(er) formats [2 vs. 2 (30), 3 vs. 3 (29)] compared with big(ger) ones, like 3 vs. 3 (30), 4 vs. 4 (30) and 6 vs. 6 (31) and 9 vs. 9 (31).

Evidence opposing this trend is limited. However, Casamichana et al. (31) did not find any statistical differences in HR measures between MM and non-MM conditions in SSG involving 3 vs. 3, 6 vs. 6, and 9 vs. 9 formats. Similarly, Sampaio et al. (29) found no significant differences in average HR and average HR% between defensive man-to-man marking and regular drills in 2 vs. 2 and 3 vs. 3 games.

### Man marking on physical parameters

Man-marking increases the external load (e.g., total distance covered, high-intensity running distance etc.) experienced by soccer players, resulting in greater overall physical demands and faster game tempo during SSG. Studies (*n* = 4; 58 players) have reported significant increases in total distance covered (26, 30, 31), average speed (30), relative total distance covered (m/min) (30), sprint distance (30), distance covered >18 km/h (26, 30), and distance covered between 13–17.9 km/h (26). In addition, significantly greater volumes of walking (27), lateral and backwards running (27), player load as measured by accelerations across intensity bands (31), and work-to-rest ratio (31) have also been observed in MM compared to non-MM formats.

Similar to the physiological variables, the effect of MM on physical variables appears to depend on player numbers, at least for certain variables (30). While low-intensity variables [walking (0–6.9 km/h), jogging (7.0–13.0 km/h)] were not statistically different between small(er) (2 vs. 2; 3 vs. 3) and big(ger) (4 vs. 4) games. Variables that indicate greater physical load, such as distance covered at striding (13.1–17.8 km/h) and high-intensity (17.9–21 km/h) speeds were greater in the smaller formats (2 vs. 2 and 3 vs. 3) using MM but not in the larger formats (4 vs. 4). Similarly, average striding speed was different for the 2 vs. 2 and 3 vs. 3 compared to the bigger format (4 vs. 4) (30). Furthermore, Casamichana et al. (31) also reported significantly greater work-to-rest ratio during MM in small (3 vs. 3) and medium (6 vs. 6) formats, but not in the largest (9 vs. 9).

### Pressure on the ball

Applying defensive pressure on the ball has become a growing topic of interest in soccer research (32–35). Studies have shown that successful defensive plays are characterized by greater defensive pressure compared with unsuccessful ones (32), supporting its value as a key performance indicator in defensive performance (32). Indeed, Tenga et al. (36) demonstrated that significantly fewer score-boxed (entry into the box with a specific outcome) were scored against a ‘tight’ defensive pressure, compared to ‘mixed’, or ‘loose’ pressure, indicating its effectiveness in limiting opposition attacks. The physical demands associated with pressing behaviour also vary according to the location and intensity of the press. Teams employing a high-press, compared with those defending in a deeper position, demonstrate increased demands in terms of total distance (TD), running distance (14,4–19,8 km/h), high-speed running distance (19,8–25,2 km/h), and the number of high-speed running efforts (34). In contrast, defending in a low block is associated with greater walking distance (<2 km/h), while mid-block defensive structures elicit demands in both running and high-speed running distances (34).

Cihan et al. (26) developed an experimental approach to assess pressure on the ball during SSG. In this protocol, two designated players were required to apply immediate defensive pressure on the ball carrier. Each team was assigned blue, red, and yellow training vest, and players were paired with players wearing different colored vests. When a blue player had the ball, the corresponding red and yellow players immediately applied defensive pressure, while the other red and yellow teammates applied defensive pressure to the free blue player, potentially creating a 2-v-4 situation. When possession changed, the colour assignments reversed accordingly, ensuring continuous defensive engagement and realistic pressure scenarios (26).

Given the clear tactical relevance of defensive pressure, coaches frequently incorporate pressing tasks into SSG to replicate match-like demands and improve defensive responsiveness. Pressure on the ball has been implemented in both youth (8) and adult (26, 37) populations using various formats, including 2 vs. 2 (8), 3 vs. 3 (26), 4 vs. 4 (37) and 8 vs. 8 (37). Typically, players are instructed to close down the space within approximately 1.5 m of the ball carrier as quickly and aggressively as possible (8), thereby promoting rapid defensive transitions and improving collecting defensive coordination.

### Pressure on the ball on physiological parameters

Applying pressure on the ball markedly increases the physiological intensity of SSG, with studies (*n* = 2; 72 players) reporting higher mean HR, percentage of time spent in 90%–100% HRmax, and blood lactate acid concentration, alongside a lower percentage of time spent in 80%–90% HRmax under pressing conditions (8). A double pressure protocol, where two players simultaneously press the ball carrier, further increased higher blood lactate concentration, mean HR, mean %HRmax, and session-RPE responses compared to free play (26). Player number appears to moderate these responses, as HR values during 8 vs. 8 with pressing are comparable to those in 4 vs. 4 with and without goalkeepers, whereas 8 vs. 8 free-touch and non-pressing conditions yield lower HR values (37). Taken together, these findings indicate that pressing substantially elevates internal load [i.e., psychobiological stress (38)] in SSG and that increasing the intensity or frequency of pressure further amplifies cardiovascular and metabolic stress.

### Pressure on the ball on physical parameters

Pressing the ball increases the external load of SSG. Studies (*n* = 2; 74 players) report a higher number of high-speed runs (20–25 km/h), more direction changes, and greater high-speed running distance (20–25 km/h) under pressing conditions, whereas distance covered at moderate speeds (15–20 km/h) was lower (8). In a double-pressure protocol, players covered greater distances in the high-intensity running zone (>18 km·h-1) compared to free play (26). These findings indicate that increasing the intensity or frequency of pressure elevates the mechanical demands of SSG.

### Pressure on the ball on tactical parameters

Evidence for tactical adaptations under pressure on the ball is limited, with only two studies and 96 players available (8, 39). Chen et al. (8) reported significantly more ball recoveries under pressing compared to free play. Rochael et al. (39) found that time pressure resulted in significant lower decision making and execution with a lower spatial exploration index. Albeit limited, these findings suggest that pressing can facilitate quicker regain of possession but may constrain exploratory behaviour and impair decision quality, indicating a potential trade-off that coaches should consider when designing SSG.

### Offside vs. no offside

Studies (*n* = 4; 96 players) utilizing offside versus no offside conditions were solely implemented in youth cohorts (40–43). SSG formats such as 4 vs. 4 (41–43) and 6 vs. 6 (40) were used.

### Offside on physiological parameters

To the authors’ knowledge, only one scientific investigation has examined offside versus no-offside in relation to physiological responses. Custodia et al. (43) reported that enforcing the offside rule did not alter physiological parameters such as peak and mean HR in a 3 vs. 3 SSG with a goalkeeper. These findings suggest that, in this format, the offside rule may not materially affect internal load; however, the evidence is limited to a single investigation and a specific game configuration, so broader generalisation is premature.

### Offside on physical parameters

Enforcing the offside rule has been found to reduce external load in SSG. Studies (*n* = 2; 48 players) report lower total distance covered (40, 41) and average speed (41), less distance at cruising speeds [13.0–16.0 km/h (40), 13.0–17.9 km/h (41)], less distance at jogging speeds (7.0–12.9 km/h) and greater distance standing/walking (0.0–6.9 km/h) under offside conditions (41). Pitch size appears to moderate these effects, with significant differences observed only on the largest pitch size of 75 m^2^ per player, whereas the smaller pitch sizes of 25 m^2^ and 50 m^2^ per player showed no significant differences (40). These findings suggest that offside constraints can diminish running volume, particularly when space per player is large enough to allow attacking depth and manipulation of the offside line. However, they should be interpreted with caution because the evidence comes from only a small number of studies.

### Offside on tactical parameters

Only a single study was found that examined the effects of the offside rule on tactical parameters during SSG. Praca et al. (42) reported that enforcing the offside rule decreased players’ in-depth positioning (length), the length-per-width ratio, and limited players’ spatial exploration during 3 vs. 3 SSG with a goalkeeper among U17 national players (42). These findings suggest that implementing the offside rule results in a more compact team shape, but should be interpreted cautiously because the evidence comes from a single study and format configuration.

## Conclusion

Changing task-constraints during SSG is an effective way to modulate training intensity. Across the available studies, MM and pressure on the ball consistently increased internal load, reflected in higher average % HR and time spend >80% maximum HR, and higher session RPE. Similarly, MM and pressure on the ball increased external load, with greater total distance, relative total distance, and more high-speed running (velocity bands at high-intensity >13 km/h) and sprint running compared with non-MM and no pressure on the ball. Evidence for tactical adaptations is absent for MM and limited for pressure on the ball, although pressing has been linked to more frequent ball recoveries and to reductions in decision quality and spatial exploration index. Offside did not meaningfully impact physiological measures in the single study identified, but it reduced physical parameters, such as total distance and low(er) intensity running (e.g., cruising and jogging 7–16 km/h). One scientific study indicated that implementing offside rules produced a more compact team structure by decreasing players’ in-depth positioning, lowering the length-per-width ratio, and decreasing the players’ spatial exploration index. Overall, these findings suggest that MM and pressure on the ball to increase internal and external load of SSG. Offside, however, reduces physical efforts and restricts tactical behaviour of players. However, these patterns should be interpreted cautiously given the limited and heterogeneous evidence. More precisely, this mini-review utilized all available scientific evidence regarding the effects of MM, pressure on the ball and offside and their effect on physiological, physical, technical and tactical parameters disregarding various task constraints in the utilized games throughout the scientific literature. Task constraints are rules and regulations such as relative pitch size (3), number of players (44), touch restrictions (4), and the presence of goalkeepers (5) that influence the aforementioned parameters of players and consequently need to manipulated carefully to account for the desired training emphasis. In addition, the statements within each section (MM, pressure on the ball and offside) might be based on different populations (youth and adults) with various playing experience and skill level. However, due to the limited available research with regards to MM, pressure on the ball and offside this mini-review has pooled the scientific research with regards to each topic. Consequently, more research using more format configurations (e.g., relative pitch size, number of players) in various populations (e.g., male, female, youth and adults) is required to provide more generalisable recommendations for coaches. Nevertheless, in the light of these limitations, coaches can utilize MM, pressure on the ball and offside to manipulate the physiological, physical, technical and tactical parameter during SSG.

## References

[1] de Paula Amorim Borges E Praça GM Figueiredo LS Vieira CA, De Conti Teixeira Costa G. Promoting tactical-technical actions during small-sided soccer games: a narrative review on constraints’ manipulation within ecological teaching models. Retos. (2022) 45:566–575. 1579-1726

[2] Clemente FM Martins FM Mendes RS. Developing aerobic and anaerobic fitness using small-sided soccer games: methodological proposals. Strength Cond J. (2014) 36(3):76–87. 10.1519/SSC.0000000000000063

[3] Rumpf MC Jager J Clemente FM Altmann S Lochmann M. The effect of relative pitch size on physiological, physical, technical and tactical variables in small-sided games: a literature review and practical guide. Front Sports Act Living. (2025) 7:1592536. 10.3389/fspor.2025.1592536 40395810 PMC12089100

[4] Rumpf MC Jäger J Altmann S Lochmann M. Touch restriction during small-sided games in soccer—effects on physiological, physical and technical and tactical performance. Front Sports Act Living. (2025) 7:1705921. 10.3389/fspor.2025.1705921 41163665 PMC12558969

[5] Rumpf MC Lochmann M. Commentary on the utilizing of goalkeepers in small-sided-games in soccer. Germ J Exerc Sport Res. (2025). 10.1007/s12662-025-01049-6

[6] Sarmento H Clemente F Harper L da Costa I Owen A Figueiredo A. Small sided games in soccer – a systematic review. Int J Perform Anal Sport. (2018) 18(5):693–749. 10.1080/24748668.2018.1517288

[7] Halouani J Chtourou H Gabbett T Chaouachi A Chamari K. Small-sided games in team sports training: a brief review. J Strength Cond Res. (2014) 28(12):3594–618. 10.1519/JSC.0000000000000564 24918302

[8] Chen X Zheng R Xiong B Huang X Gong B. Comparison of the physiological responses and time-motion characteristics during football small-sided games: effect of pressure on the ball. Front Physiol. (2023) 14:1167624. 10.3389/fphys.2023.1167624 37275220 PMC10235494

[9] Clemente FM Afonso J Sarmento H. Small-sided games: an umbrella review of systematic reviews and meta-analyses. PLoS One. (2021) 16(2):e0247067. 10.1371/journal.pone.0247067 33577611 PMC7880470

[10] Plakias S Kasioura C Pamboris GM A grounded theory for professional soccer teams’ playing styles: towards a consensus. Int J Sports Sci Coach. (2025) 30(1):159–172. 10.1177/17479541241300605

[11] Sporis G Samija K Vlahovic T The latent structure of soccer in the phases of attack and defense. Coll Antropol. (2012) 36(2):593–603. 22856250

[12] Plakias S Moustakidis S Kokkotis C Identifying soccer players’ playing styles: a systematic review. J Func Morph Kines. (2023) 8(104):15. 10.3390/jfmk8030104 PMC10443261 37606399

[13] Andrienko G Andrienko N Burch M Weiskopf D. Visual analytics methodology for eye movement studies. IEEE Trans Vis Comput Graph. (2012) 18(12):2889–98. 10.1109/TVCG.2012.276 26357198

[14] Bortnik L Burger J Rhodes D. The mean and peak physical demands during transitional play and high pressure activities in elite football. Biol Sport. (2022) 39(4):1055–1064. 10.5114/biolsport.2023.112968 36247966 PMC9536371

[15] Nassis GP Massey A Jacobsen P Elite football of 2030 will not be the same as that of 2020: preparing players, coaches, and support staff for the evolution. Scand J Med Sci Sports. (2020) 30(6):962–964. 10.1111/sms.13681 32424904

[16] Wright C Atkins S Polman R Jones B Sargeson L. Factors associated with goals and goal scoring opportunities in professional soccer. Int J Perform Anal Sport. (2011) 11(3):438–449. 10.1080/24748668.2011.11868563

[17] Maruenda FB. Can the human eye detect an offside position during a football match? Br Med J. (2004) 329(7480):1470-2. 10.1136/bmj.329.7480.1470 15604187 PMC535985

[18] Catteeuw P Gilis B Jaspers A Wagemans J Helsen W. Training of perceptual-cognitive skills in offside decision making. J Sport Exerc Psychol. (2010) 32(6):845–61. 10.1123/jsep.32.6.845 21282841

[19] Wühr P Furley P Mertes M Memmert D. A home advantage in offside decisions in German professional football (soccer). Collabra: Psychology. (2025) 11(1):1–16. 10.1525/collabra.133671

[20] Zhao Y. Downtrends in offside offenses among ‘the big five’ European football leagues. Front Psychol. (2021) 12:719270. 10.3389/fpsyg.2021.719270 34616335 PMC8488103

[21] Catteeuw P Gilis B Garcia-Aranda JM Tresaco F Wagemans J Helsen W. Offside decision making in the 2002 and 2006 FIFA world cups. J Sports Sci. (2010) 28(10):1027–32. 10.1080/02640414.2010.491084 20686996

[22] Lago-Penas C Lago-Ballesteros J Dellal A Gomez M. Game-related statistics that discriminated winning, drawing and losing teams from the Spanish soccer league. J Sports Sci Med. (2010) 9:288–293. PMC3761743/pdf/jssm-09-288.pdf 24149698 PMC3761743

[23] Prieto-Gonzalez P Martin V Sal-de-Rellan A. Impact of defensive team variables on goals conceded in the first division of the Spanish soccer league: a 10-year study. Biol Sport. (2025) 42(3):17–28. 10.5114/biolsport.2025.145914 40656987 PMC12244413

[24] Plakias S Armatas V Giakas G. Technical–tactical analysis of corner kicks in male soccer: a systematic review. Appl Sci. (2025) 15:4984. 10.3390/app15094984

[25] Gerisch G Rutemölller E Weber K. Sport Medical Measurements of Performance in Soccer. London: E & FN Spon (1988).

[26] Cihan H. The effect of defensive strategies on the physiological responses and time-motion characteristics in small-sided games. Kinesiol. (2015) 47(2):179–187. 796.332.015:611

[27] Aroso J Rebelo AN Gomes-Pereira J. Physiological impact of selected game-related exercises. J Sports Sci. (2004) 22(6):522. 10.1080/02640410410001675432

[28] Ngo JK Tsui M-C Smith AW Carling C Chan G-S Wong d. The effects of man-marking on work intensity in small-sided soccer games. J Sports Sci Med. (2012) 11:109–114. PMC3737838 24149127 PMC3737838

[29] Sampaio J García G Maçãs V Ibáñez S Abrantes C Caixinha P. Heart rate and perceptual responses to 2×2 and 3×3 small-sided youth soccer games. J Sports Sci Med. (2007) 6:121–122.

[30] Aasgaard M Kilding AE. Does man marking influence running outputs and intensity during small-sided soccer games? J Strength Cond Res. (2020) 34(11):3266–3274. 10.1519/JSC.0000000000002668 33105379

[31] Casamichana D Roman-Quintana JS Castellano J Calleja-Gonzalez J. Influence of the type of marking and the number of players on physiological and physical demands during sided games in soccer. J Hum Kinet. (2015) 47:259–68. 10.1515/hukin-2015-0081 26557209 PMC4633261

[32] Forcher L Forcher L Altmann S Jekauc D Kempe M. The keys of pressing to gain the ball – characteristics of defensive pressure in elite soccer using tracking data. Sci Med Foot. (2024) 8(2):161–169. 10.1080/24733938.2022.2158213 36495564

[33] Fernandez-Navarro J Ruiz-Ruiz C Zubillaga A Fradua L. Tactical variables related to gaining the ball in advanced zones of the soccer pitch: analysis of differences among elite teams and the effect of contextual variables. Front Psychol. (2019) 10:1–14. 10.3389/fpsyg.2019.03040. 32038403 PMC6985566

[34] Plakias S Tsatalas T Moustakidis S Exploring the influence of playing styles on physical demands in professional football. Hum Mov. (2023) 24(4):36–43. 10.5114/hm.2023.133919

[35] Kweir AN Al-Khadum I. The effect of defensive tactics exercises based on the high-press strategy on developing some basic skills and motor response speed in youth football. Modern Sport. (2024) 23(1):10. 10.54702/cm6eh987

[36] Tenga A Holme I Ronglan LT Bahr R. Effect of playing tactics on goal scoring in Norwegian professional soccer. J Sports Sci. (2010) 28(3):237–44. 10.1080/02640410903502774 20391095

[37] Sassi A Reilly T Impellizzeri F. A comparison of small-sided games and interval training in elite professional soccer players. J Sport Sci. (2004) 22:562.

[38] Ide BN Silvatti AP Staunton CA Marocolo M Oranchuk DJ Mota GR. External and internal loads in sports science: time to rethink? Int J Strength Cond. (2023) 3(1):1–8. 10.47206/ijsc.v3i1.234

[39] Rochael M Praca GM. Designing small-sided games for counterattack training in youth soccer. Int J Sports Sci Coach. (2024) 19(2):687–697. 10.1177/17479541231170830

[40] Castillo D Raya-Gonzalez J Manuel Clemente F Yanci J. The influence of offside rule and pitch sizes on the youth soccer players’ small-sided games external loads. Res Sports Med. (2020) 28(3):324–338. 10.1080/15438627.2020.1739687 32183556

[41] Custodio IJO Praca GM Paula LV Bredt SDGT Nakamura FY Chagas MH. Intersession reliability of GPS-based and accelerometer-based physical variables in small-sided games with and without the offside rule. Proc Inst Mech Eng Pt P J Sports Eng Tech. (2022) 236(2):134–42. 10.1177/1754337120987646

[42] Praca GM Chagas MH Bredt SGT Andrade AGP Custodio IJO Rochael M. The influence of the offside rule on players’ positional dynamics in soccer small-sided games. Sci Med Footb. (2021) 5(2):144–149. 10.1080/24733938.2020.1819559 35077330

[43] Custodio IJO Dos Santos R de Oliveira Ildefonso R Effect of small-sided games with and without the offside rule on young soccer players: reliability of physiological demands. Int J Environ Res Public Health. (2022) 19(17):10544. 10.3390/ijerph191710544 36078260 PMC9518382

[44] Castellano J Casamichana D Dellal A. Influence of game format and number of players on heart rate responses and physical demands in small-sided soccer games. J Strength Cond Res. (2013) 27(5):1295–303. 10.1519/JSC.0b013e318267a5d1 22836601

